# A Birth Cohort Analysis to Study Dog Walking in Adolescence Shows No Relationship with Objectively Measured Physical Activity

**DOI:** 10.3389/fvets.2017.00062

**Published:** 2017-05-16

**Authors:** Carri Westgarth, Andrew R. Ness, Calum Mattocks, Robert M. Christley

**Affiliations:** ^1^Department of Epidemiology and Population Health, Institute of Infection and Global Health, University of Liverpool, Neston, UK; ^2^Istitute of Veterinary Science, University of Liverpool, Neston, UK; ^3^National Institute for Health Research (NIHR), Biomedical Research Unit in Nutrition, Diet and Lifestyle at the University Hospitals Bristol NHS Foundation Trust, The University of Bristol, Bristol, UK; ^4^School of Oral and Dental Sciences, University of Bristol, Bristol, UK; ^5^Institute of Public Health, University of Cambridge School of Clinical Medicine, Cambridge, UK

**Keywords:** Avon Longitudinal Study of Parents and Children, exercise, dogs, walking, adolescent, child, physical activity

## Abstract

Physical inactivity during childhood and adolescence is a serious health concern. There are few studies of the activity undertaken by adolescents when walking with the family dog, and the effect of this on objectively measured physical activity levels. Objective measures of physical activity using accelerometers were recorded at age 11–12, 13–14, and 15–16 years in the Avon Longitudinal Study of Parents and Children (ALSPAC) (ALSPAC, UK) birth cohort during the 2000s. Family pet ownership was collected retrospectively using a questionnaire at age 18 years, for the ages 7, 11, 13, and 15 years. In addition, approximate frequency per week of walks undertaken with dogs were also reported. Multilevel, multivariable modeling was used to investigate the relationship between dog ownership and dog walking status, and physical activity outcomes. There were a total of 4,373 complete data observations for use in 2,055 children. Reported participation in dog walking tended to increase during adolescence, as did dog ownership. The majority of who own dogs reported walking them either 2–6 times/week (range 39–46%) or never (range 27–37%). A small minority (7–8%) reported walking their dog every day. Most reported never walking any other dog either (94–87%). We found no evidence for an association between dog ownership or reported dog walking, and objectively measured physical activity (counts per minute, *P* = 0.3, or minutes of moderate-to-vigorous physical activity, *P* = 0.7) during adolescence. This study provides no evidence to support a relationship between adolescent dog ownership and physical activity, and demonstrates the importance of using objective activity measures and considering dog walking rather than just dog ownership.

## Introduction

Physical activity is important for optimal health and the prevention of chronic diseases; however, the proportion of children (5–15 years) meeting guidelines (minimum 1 h/day of moderate activity) is low (21% boys and 16% girls) (1). Therefore, it is crucial to gather evidence of effective intervention means that increase physical activity. Adults who own dogs have been shown to be more physically active than those who do not own dogs (2). Further, owners who walk their dogs regularly may also have lower weight status (3). However, the benefit of dog walking for children and adolescents is less clear. This target group is particularly important given the increasing prevalence of childhood obesity and low levels of physical activity.

Two Australian cross-sectional studies (one self-reported, one objective accelerometer-measured) and one UK cross-sectional (accelerometer) study have demonstrated a small positive association between dog ownership and physical activity in children (4–6). However, a further US self-report study showed no evidence of an association in 4- to 10-year olds (7). One cross-sectional study also found evidence of some positive association between dog ownership and objectively measured physical activity in adolescents (8), however, another using diary reports found no association (9). In summary, previous studies have been limited to cross-sectional data and have used mainly self-reported as opposed to objective measures of physical activity with very little research on the adolescent age group.

Further, no previous analyses of child/adolescent physical activity outcomes have accounted for reported dog walking specifically, which has been shown to be a key concerning increased physical activity levels in adults, rather than ownership (2). In fact, very few studies have actually examined the extent of involvement of young people in dog walking (5, 10, 11).

In summary, there are no studies of the role adolescents take in walking with the family dog, and the effect of this on objectively measured physical activity. This study aims to fill this gap using longitudinal data from a well-characterized UK birth cohort. The objective of this study was to examine the association between dog ownership and involvement in dog walking with objectively measured physical activity during adolescence. We hypothesized that adolescents who reported walking their dogs would have higher physical activity levels than those who did not own a dog, or did but did not walk it. We also hypothesized that a dose–response effect would be seen with more frequent dog walking associated with increasing levels of activity.

## Materials and Methods

### Data Collection

The Avon Longitudinal Study of Parents and Children (ALSPAC) is a prospective study, described in full elsewhere (12), which recruited 14,541 pregnant women resident in Avon, UK, with expected dates of delivery between 1st April 1991 and 31st December 1992. Of the initial 14,541 pregnancies, all but 69 had a known birth outcome and, of these, 195 were twin, three were triplet, and one was a quadruplet pregnancy meaning that there were 14,676 fetuses in the initial ALSPAC sample; 14,062 were live births and 13,988 were alive at 1 year. At approximately 7 years, a further enrollment phase added more children. The total sample size for analyses using any data collected after the age of seven is, therefore, 15,247 pregnancies, resulting in 15,458 fetuses. Of this total sample of 15,458 fetuses, 14,775 were live births and 14,701 were alive at 1 year of age. The study website contains details of all the data that is available through a fully searchable data dictionary (http://www.bris.ac.uk/alspac/researchers/data-access/data-dictionary/). Ethical approval for the study was obtained from the ALSPAC Law and Ethics Committee and the Local Research Ethics Committees and the participants provided written informed consent.

Objective measures of physical activity using Actigraph accelerometers were recorded at age 11–12, 13–14, and 15–16 years and have been described in detail elsewhere (13). Children were asked to wear an Actigraph accelerometer on their right hip for 7 days; data were valid if the children had worn it for at least 10 h/day for 3 days. Outcomes recorded were average counts per minute (CPM) of overall physical activity per day, and average minutes per day spent in moderate-to-vigorous physical activity (MVPA) using an Actigraph cut point of >3,600 CPM as previously developed and validated on a subsample (14).

Family pet ownership information was collected retrospectively at age 18, for the ages 7, 11, 13, and 15 years, by questionnaire survey. Participants were asked whether they had any pets in their household when they were that age and how many cats, dogs, rabbit, rodents, birds, fish, tortoises/turtles, and horses. In addition, approximate frequency per week of walks undertaken with the pet dog were also reported, as were approximate frequency per week of walks undertaken with any other dog (e.g., belonging to a friend or family member). At age 13–14 years, children were asked to complete a computer-based activity recall session indicating activities that occurred on the previous day, which included walking the dog (15).

### Data Analysis

There were a total of 4,373 complete data observations for use in 2,055 children (age 11–12 years had 1,821; 13–14 years had 1,547; and 15–16 years had 1,005). Five-hundred eight children were observed at one time point only, 776 twice, and 771 at all three time periods.

For each time point, the variables of dog ownership (yes/no) and of reported frequency of dog walking were further categorized into a combined dog ownership/walking variable: non-dog owner; never walks dog, walks dog once a week, walks dog 2–6 times/week, or walks dog 7 or more times a week. Non-dog owners comprised 3,214 (73.5%) observations, dog owners who walked 0/week 286 (6.5%), 1/week 258 (5.9%), 2–6/week 531 (12.1%), and ≥7/week 84 (1.9%) of observations.

The association of dog walking with CPM and MVPA were assessed using random effects linear regression models in order to account for clustering of data within individuals across all three time points. The outcome MVPA was skewed and so was logged (log_10_) prior to analysis. Variables considered as potential confounders included: age at physical activity data collection (days), gender, season of data collection (months), maternal social class by occupation, and maternal education level at gestation.

Initially, for each outcome, all variables were compared using univariable random effects models. Linearity of the relationship between continuous variables and the outcomes was assessed using GAM models (mgcv package in R). For each analysis (CPM and MVPA), datasets that only included variables with data for the outcome and all input variables were constructed. In all cases, the form of the relationship was considered suitable to be modeled as linear.

For each outcome, model building commenced with construction of a maximal model that included the main dog ownership/walking explanatory variable and all potential confounders. In addition, two- and three-way interactions between dog walking, season, and gender were assessed. Because of considerable collinearity between maternal education and maternal SES, only maternal education was considered in the maximal model. Subsequently, a backward elimination procedure was used with the significance of each term assessed by evaluating the change in deviance (LRT) associated with their removal from the model. The main variable of interest, dog walking, was retained in the final model irrespective of its significance. Model fit was assessed by visual examination of residuals against predicted values. All analyses were undertaken using the *R* language for statistical computing using the lmer function, in the lme4 package. Due to the complexity of the novel analysis method, sample size calculations could not be performed.

## Results

### Pet Ownership and Role in Dog Walking

Age 7 pet ownership collected retrospectively was highly associated with pet ownership reported by the carers at the time the child was age 7 (*P* < 0.0001), suggesting accurate recall. Reported pet and dog ownership, and frequency of participation in dog walking, across all four retrospective and one current time points is reported in Table 1. Reported participation in dog walking tended to increase during adolescence, as did dog ownership. The majority of adolescents who own dogs reported walking them either 2–6 times/week (range 39–46%) or never (range 27–37%). A small minority (7–8%) reported walking with their dog every day. Most reported never walking any other dog either (87–94%) (Table 1). In the activity-recall coding of the previous day’s activities at age 13, 510 (8.9%) reported that they had walked a dog.

**Table 1:** **Retrospective reporting (at age 18 years) of pet ownership and dog walking at age 7, 11, 13, 15, and 18 years**.

		Retrospective				Current
		7 years	11 years	13 years	15 years	18 years
	
		*n* (%)	*n* (%)	*n* (%)	*n* (%)	*n* (%)
Pet	No	714 (23.1)	647 (21.1)	727 (23.5)	783 (25.8)	854 (27.6)
	Yes	2,732 (76.9)	2,416 (78.9)	2,333 (76.5)	2,251 (74.2)	2,244 (72.4)
Dog	No	2,357 (76.4)	2,215 (72.3)	2,122 (69.5)	1,996 (65.8)	1,965 (63.4)
	Yes	729 (23.6)	848 (27.7)	931 (30.5)	1,039 (34.2)	1,136 (36.63)
Freq dog walks own dog	Never	432 (48.4)	351 (36.8)	314 (31.3)	298 (27.3)	395 (33.0)
	Once a week or less	124 (13.9)	161 (16.9)	202 (20.1)	200 (18.3)	246 (20.6)
	2–6/week	290 (32.5)	376 (39.4)	417 (41.5)	504 (46.1)	451 (37.7)
	7/week+	46 (5.2)	66 (6.9)	71 (7.1)	91 (8.3)	105 (8.8)
Freq dog walks any other dog	Never	1,769 (93.6)	1,782 (92.2)	1,800 (90.4)	1,820 (88.5)	1,835 (86.8)
	Once a week or less	71 (3.8)	85 (4.4)	89 (4.5)	128 (6.2)	148 (7.0)
	2–6/week	42 (2.2)	56 (2.9)	86 (4.3)	97 (4.7)	115 (5.4)
	7/week+	8 (0.4)	10 (0.5)	16 (0.8)	12 (0.6)	16 (0.8)

*Data collected for the 2000s in the Avon Longitudinal Study of Parents and Children (ALSPAC), UK*.

### Counts per Minute

The final model for CPM (Table 2) included dog walking frequency, gender, month, age, and maternal education level. There was no evidence of a difference among participants with different dog walking frequencies (*P* = 0.3). Despite this, there appeared to be a tendency among dog owners toward increasing CPM as dog walking frequency increased.

**Table 2:** **Association between dog ownership/dog walking and counts per minute (CPM) of physical activity in adolescence and log_10_[moderate-to-vigorous physical activity (MVPA)] in adolescence (4,373 observations in 2,055 children)**.

	Unadjusted estimate	Unadjusted CI	Adjusted^a^ estimate	Adjusted^a^ CI	*P*
**CPM**						
(Intercept)	534.44	527.25–541.64	902.12	860.71–943.53	
Dog ownership/walking					0.3
Non-dog owner	Ref		Ref		
Never	−14.00	−36.20–8.20	−3.80	−24.16–16.57	
Once a week or less	−23.67	−46.60–0.74	−0.13	−21.18–20.92	
2–6/week	−9.60	−26.94–7.80	9.01	−6.99–25.02	
7/week+	21.69	−17.33–60.72	35.14	−30.22–12.29	
**MVPA**						
(Intercept)	1.25	1.24–1.27	1.43	1.36–1.52	
Dog ownership/walking					0.7
Non-dog owner	Ref		Ref		
Never	−0.03	−0.08–0.01	−0.02	−0.07–0.02	
Once a week or less	−0.02	−0.06–0.02	−0.00	−0.04–0.04	
2–6/week	−0.02	−0.05–0.02	−0.01	−0.04–0.03	
7/week+	0.03	−0.04–0.11	0.03	−0.04–0.10	

*Data collected during the 2000s in the Avon Longitudinal Study of Parents and Children, UK*.

*^a^Adjusted for month, gender, age, and maternal education. Observation point set as level 1 and child as level 2 in hierarchical model, as children provided data from approximately ages 11, 13, and 15*.

### Moderate-to-Vigorous Physical Activity

The final adjusted model for MVPA (Table 2) demonstrated no evidence of an association between dog ownership/walking and level of MVPA (*P* = 0.7). In fact, only the most frequent dog walkers even had MVPA estimates above those of non-dog owners.

## Discussion

We found no evidence of an association between dog ownership or reported role in dog walking and objectively measured physical activity during adolescence. This suggests that family dog walking during adolescence is low and does not impact on physical activity levels. Our findings are in line with those of Mathers et al. (9) who found no association between dog ownership or time spent playing/caring for pets and physical activity calculated *via* a self-reported diary. In regards to MVPA, our findings also agree with the only other study of dog ownership using objectively measured PA in adolescents, although they did find a small association with CPM (8). There are no previous studies detailing the role of adolescents in dog walking activities; however, only 7–8% reported walking approximately daily with the dog compared to 35% in 9- to 10-year olds (11). Previous studies suggest that involvement in pet dog walking may decrease as a child gets older (4–6); however, our data showed that reported dog walking increased at least through adolescence, both for with their own dog or someone else’s dog.

This study has a number of strengths compared to previous studies. It uses a large dataset from a well-characterized UK birth cohort, including objectively measured physical activity outcomes. The predictor variable consisted of frequency of walking with the dog, not simply ownership or time spent with it, and adjustment for key confounding variables was performed. The model used allowed ownership and dog walking to vary across observation time points for each child that contributed to the analysis of overall effect of dog ownership/walking at the observation level. In addition, although we did not interpolate missing data, the use of multilevel modeling does have the advantage of enabling incorporation of the data for each child for each time point. Hence, if a child only had data for some but not all time points that would still be included in analysis, maximizing data usage.

There are some limitations, in that, the ownership and dog walking frequency data were estimated retrospectively rather than concurrently, although, a previous study has shown that recall of childhood pet ownership by young adults is accurate (16). In addition, we tested recall accuracy in our dataset for age 7 and the findings were consistent. Therefore, it is likely that dog ownership recall is accurate, and that previous dog walking habits are likely to be recalled with reasonable accuracy. Further, no data were collected regarding the type of dog owned. For example, size of the dog can influence how often it is walked (17). As our independent variable included reported dog walking frequency, this should not overly affect our results. However, smaller dogs that are walked may plausibly be walked shorter distances, leading to less physical activity recorded, and our study could not examine this. This survey only examined frequency, not length of dog walks, and also only examined dog walking, not other physical activity that might result from owning a pet dog such as playing or caring for them. However, the frequency of participation in dog walking is likely the primary influence of the dog on physical activity of dog owning children (6). The effect of dog ownership on physical activity in children besides dog walking such as active play requires further investigation.

In conclusion, we found no evidence of an association between dog ownership or walking and physical activity in adolescence. This study used objectively measured physical activity rather than self-report and highlights the importance of assessing dog walking directly rather than using dog ownership as a proxy. Future cohort studies should collect more detailed information about interactions with pets if analysis of the effects of pet ownership on human health is to be worthwhile, including detail on frequency, duration, and distance of walking with the pet dog, preferably using objective measures. Given that child involvement in dog walking has been shown to be associated with the strength and type of relationship with the dog (11), measures of attachment to the pets should also be studied.

## Availability of Data and Materials

The dataset(s) supporting the conclusions of this article are available via application to the ALSPAC Executive Committee.

## Ethics Statement

This study was carried out in accordance with the recommendations of the ALSPAC Law and Ethics Committee and the Local Research Ethics Committees with written informed consent from all subjects. All subjects gave written informed consent in accordance with the Declaration of Helsinki. Ethical approval for the study was obtained from the ALSPAC Law and Ethics Committee and the Local Research Ethics Committees.

## Author Contributions

CW designed the pet ownership data collection. CW and RC designed the analysis, conducted the analysis, and drafted the paper. AN and CM collected the physical activity data, assisted with the analysis, and commented on the manuscript.

## Disclaimer

This publication is the work of the authors and CW and RC will serve as guarantors for the contents of this paper. The views expressed are those of the author(s) and not necessarily those of the NHS, the NIHR, the Department of Health or Public Health England.

## Conflict of Interest Statement

The authors declare that the research was conducted in the absence of any commercial or financial relationships that could be construed as a potential conflict of interest. Funding bodies had no role in design, collection, analysis, and interpretation of data; in the writing of the manuscript; or in the decision to submit the manuscript for publication.
